# The Safety of Hospital Beds: Ingress, Egress, and In-Bed Mobility

**DOI:** 10.1177/2333393615575321

**Published:** 2015-04-27

**Authors:** Janice M. Morse, Pierre Gervais, Charlotte Pooler, Andrew Merryweather, Alexa K. Doig, Donald Bloswick

**Affiliations:** 1 The University of Utah, Salt Lake City, Utah, USA; 2 University of Alberta, Edmonton, Alberta, Canada

**Keywords:** safety, patient, falls / falling, health care, long-term, nursing, gerontological, observation, risk, behaviors

## Abstract

To explore the safety of the standard and the low hospital bed, we report on a microanalysis of 15 patients’ ability to ingress, move about the bed, and egress. The 15 participants were purposefully selected with various disabilities. Bed conditions were randomized with side rails up or down and one low bed with side rails down. We explored the patients’ use of the side rails, bed height, ability to lift their legs onto the mattress, and ability to turn, egress, and walk back to the chair. The standard bed was too high for some participants, both for ingress and egress. Side rails were used by most participants when entering, turning in bed, and exiting. We recommend that side rails be reconsidered as a means to facilitate in-bed movement, ingress, and egress. Furthermore, single deck height settings for all patients are not optimal. Low beds as a safety measure must be re-evaluated.

Estimates for the number of falls in hospital range from 20% from the bed (Healey & Scobie, 2007) to approximately 60% to 70% “from bed or bedside chair” (Oliver, 2002, p. 415). This variation depends on the amount of time a patient occupies the bed (for instance, fall rates are lower if the patient is out of bed for a number of hours per day: They have less opportunity to fall from the bed). Other considerations are the patient’s condition, the quality of the nursing care (falls are now a nurse-sensitive measure; Williams, 2004), and the nurse-to-patient ratio (Dunton, Gajewski, Taunton, & Moore, 2004). Analysis of type and number of falls in a community hospital recorded 51% of falls occurred getting into or out of bed; 95% of these resulted in minor injury, and 4.95% resulted in major injury (Tzeng, 2010). However, *how* the fall was recorded alters our count of “falls from the bed.” If, for instance, the fall was recorded as “patient found on floor” and the activity was “going to the bathroom,” the fall may actually be a “getting-out-of-bed” fall, with the forward trajectory of the fall resulting in the patient found on the floor some distance from the bed. Nevertheless, although fall rates vary considerably, it is apparent that the *bed* is a major factor in patient falls. Falls occur frequently when the patient is getting into bed, is reaching from the bed, rolls out of bed, is transferring from a wheelchair, or is getting into or climbing out of bed.

Our professional response to this phenomenon is extraordinary: We continually ask the patient to use the call bell, to stay in bed until the nurse arrives, and not to get out of bed without assistance. Unfortunately, those we ask are ill, confused, overestimate their abilities, are unable to stand and walk safely, and/or need to get to the bathroom urgently. Their beds are at a substantial distance from the bathroom, and the distance from the bed to the bathroom in some institutions was measured to be as much as 16 feet, and usually without a handhold or support.

And, we still wonder why patients fall.

The responsibility for fall intervention has been placed squarely on the shoulders of nurses: We ask nurses to *observe*, yet patients are in individual rooms and out of nurses’ direct line of sight; we ask nurses to *respond* to bed alarms, yet the delay before alarm sounds (once the patient is off the bed deck) is a up to 9 seconds. Moreover, the sound of the bed alarm may be muted by ambient noise in the unit, or the nurse may be occupied with other essential tasks, hence unable to immediately rescue the patient. We ask nurses to do hourly toileting rounds (when they are caring for six or eight patients); we ask nurses to assist the patient out of bed (and injuries to nurses’ backs are at epidemic levels).

It should be noted that most of the research exploring patient falls from the bed lacks precision. Most falls are not witnessed; they are sometimes reported by patients, and sometimes reported by nurses or relatives, having heard the fall or found the patient on the floor.

The common denominator for hospital fall causation is the *bed*, and the common theme for intervention is the *nurse*. It is time for a safe environment for patients, and the first step is to determine the risks to patient safety that involves the bed.

The purpose of this project was to examine the *safety of the bed* and the patient’s ability to ingress, egress, and to move about the bed. We asked these questions:

- **Research Question 1:** How are the side rails used, and do they enable the patient to enter and exit the bed, to lie down, to sit up, and to turn over in bed more easily?
- **Research Question 2:** Is the bed height (from the floor to the top of the mattress) a factor in influencing the ease of entering and exiting the bed and the stability of the patient’s gait?
- **Research Question 3:** Can patients turn over easily and move about safely in a hospital bed?
- **Research Question 4:** Are low beds safer than a standard bed height both for ingress and egress?

## Literature Review

A preliminary review of the literature revealed that patients fall from the bed by,

1. slipping, sliding, falling, or rolling from the bed, and these falls are preventable by providing side rails, reducing the bed height (Capezuti et al., 2008), or by providing low-low beds to prevent injury (Healey & Scobie, 2007);
2. losing balance when attempting sit-to-stand from the bed, especially when the bed is too low (Capezuti et al., 2008), and these falls are preventable by increasing bed height;
3. falling, when climbing over the side rails, or over the end of the bed, and these falls are prevented by removing the side rails from the bed, or using a bed alarm, and increased surveillance (Bowers, Lloyd, Lee, Powell-Cope, & Baptiste, 2008).

This is an interesting list, because the interventions to correct the cause of one fall (e.g., reducing the height of the bed), may become the cause of another fall, with a contradictory intervention (e.g., increasing the height of the bed). What *is* the safe height of a hospital bed? Should the rails be up or down? Do bed alarms reduce fall risk? A 2012 Cochrane review exploring methods to prevent patient injuries due to falling from a bed located only two studies examining the bed (Haines, Bell, & Varghese, 2010; Tideiksaar, Feiner, & Maby, 1993), and concluded that there is a “lack of high quality evidence for or against the use of low beds, bed exit alarms, floor mats and side rails” (Anderson, Boshier, & Hanna, 2012, p. 13).

*Patient factors* that have been extensively explored are gait, strength, balance, and so forth, usually recommending exercises such as tai chi (Verhagen, Immink, van der Meulen, & Bierma-Zeinstra, 2004), but these interventions are not a “quick fix” for the hospitalized patient. Studies examining how patients climb into or out of bed are uncommon. One study exploring falls and fall injuries from the bed (Bowers et al., 2008) calculated risk of injury when falling from the bed, and over side rails, using mannequins. In summary, little is known about the safety of the hospital bed, as it is actually used.

Because our patients are ill, they may be confused, they may overestimate their abilities and may be unable to stand or walk safely, and because of the assumption that “one bed size fits all,” the safety of the hospital bed used for all patients must be examined. All patients are not of similar heights, weights, and capabilities; yet, institutional policies (i.e., “Universal fall precautions”) are the same for all patients, as are fall prevention guidelines, which recommend keeping the occupied bed in the low position (Hartford Institute for Geriatric Nursing, 2008; Tzeng et al., 2012; Quigley et al., 2009). Standard interventions are recommended for those patients scoring at medium risk, and additional interventions for high-risk scores, regardless of actual individual needs for various gaits, mental conditions, or other factors that increase (or decrease) risk of falling. Consequently, the deck height on the standard medical*-*surgical bed, when in the low position, is the same height for all patients regardless of stature, strength, gait, and stability, or other fall risk factors. Therefore, to evaluate the safety of this standard (i.e., “set the occupied bed in low position”), and how patients use side rails for mobility, we purposefully selected patients with a range of physical characteristics and disabilities, and evaluated their abilities to enter, turn, and exit from a standard medical-surgical bed in a low position (23″ mattress height) with and without side rails, and a low (15″ mattress height) bed without side rails.

## Method

### Design

This descriptive exploratory study used a one-group design, with participants randomly assigned (block design; Urbaniak & Plous, 1997–2003) to the bed and side rail position. The three conditions were a *standard medical-surgical hospital bed*^1^ in lowest position (23″ mattress height) with (a) all side rails down, (b) top side rails up and lower side rails down, and (c) a *low hospital bed* (15″ mattress height) with no side rails. Participant observational methods were used to analyze data. Participants’ macro- and micro-analytic movements were coded and recorded.

### Equipment

We tested two bed heights, 23″ mattress height with standard side rails and 15″ mattress height.^2^ A floor mat with a beveled edge, was in place for high-risk patients, until a tripping risk was observed, and the mat was subsequently discontinued for Participants 11, 13, 14, and 15 (see Doig & Morse, 2010). If the patient had a Morse Fall Scale (MFS; Morse, 2009) score of 55 or above, hip protectors were used as a safety measure.

### Setting

The study was conducted in a nursing simulation laboratory, organized for videotaping using four digital cameras. Prior to daily taping, the room was calibrated to construct a 3D grid for analysis, and recalibrated as necessary throughout the study. Markings on the floor indicated the placement of the position of the bed’s wheels, so that the beds could be interchanged between trials, and replaced in the same position.

**Figure 1.**
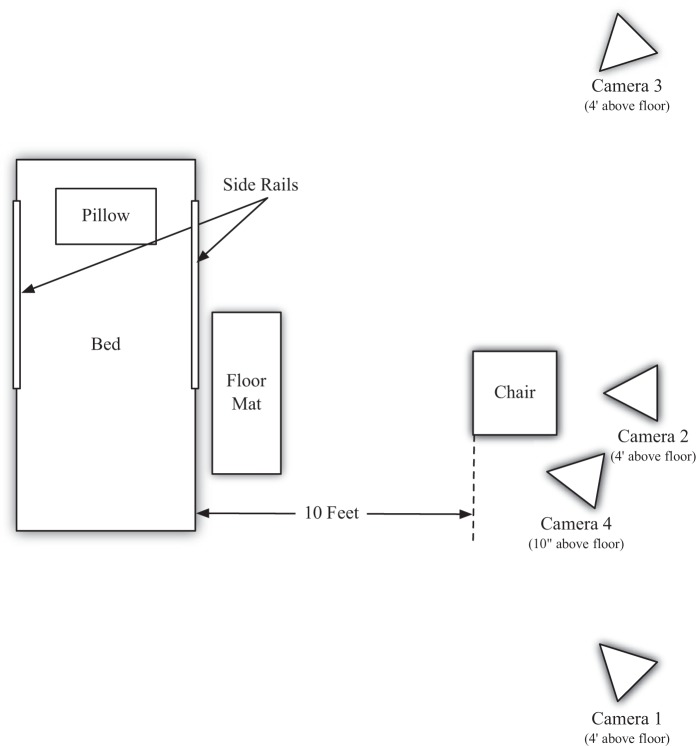
Floor plan showing placement of cameras.

Three cameras were used for taping the participants’ body movements (two lateral and one central), and the fourth camera was set at floor level, to determine how the foot struck the floor. These four cameras were synchronized by flashing a laser pointer at a target at the beginning of taping for each participant (Figure 1 for camera placement).

### Sample

The assumption underlying this study was that nursing staff had little control over adult patients’ assignment to a particular bed in a unit. As all beds are usually identical in a unit (same type, make and model), all beds therefore, must be safe for all patients. Hence, in this study, we used maximum variation sampling and purposefully selected 15 patients with a range of abilities and disabilities, as follows:

- Three surgical acute care patients with abdominal wounds, to determine the effect of pain on movement. These participants had an IV, normal gait; two with a MFS score of 20 and one with a MFS score of 35;
- Four patients with Parkinson disease (one used a cane and one used a walker), MFS score 35, 65, and two with a score of 75;
- Two patients with hemiplegia. Both used a cane and one an assist belt (MFS scores: 50 and 75, respectively);
- Three nursing home patients with a “weak and impaired” gait, two of whom used a walker (MFS scores: 40, 50, and 65); and
- Three elderly patients (MFS < 25, with a normal gait; Participants 1, 2, and 10) were used as a comparison group.

### Procedures

At the beginning of the trial, participants were seated in a chair 10 feet from the bed. They were asked to walk to the bed, to sit on the bed, and to lie down. They were then asked to turn toward the camera, and then to lie on their backs. Finally, they were asked to sit, to get out of the bed, to stand, walk back to the chair, and to sit down. This sequence was repeated for each of the three randomly assigned conditions: Standard bed with side rails, standard bed with no side rails, and low bed with no side rails. All participants were asked to perform tasks using the walking aids that they normally used.

All trials were videotaped, to macro- and micro-analyze the participants’ movements. Two researchers independently coded the videotapes, using the coding guide (see List 1).

**List 1.** Coding system.

***Standard Bed, top side rails up***
1. Subject Number
INGRESS
2. What does the patient use for support when reaching the bed?
Hands—Hand flat on the mattress
Knuckles—clenched fist on the bed?
Fingers—Hand outstretched?
3. Nurse assist patient?
No-—Unassisted
Yes—Assisted by 1 (1) or (2) RNs?
4. Move around side rail? Does the patient lift him/herself around the side rail?
Yes
No
5. Leg effort (Assist) Is the patient able to lift his/her legs onto the bed without straining?
Yes—Legs lifted easily
No—Patient had difficulty in lifting legs onto the bed
(Assist)—if yes, were the legs lifted onto the bed by the RN? Yes/no?
6. Does the patient move up in the bed (That is pushing with hands on the mattress to lift buttocks, and pushing with heals to “bounce” up bed? (Number tries)
If yes, (Number of tries)
No
IN BED MOVEMENT *Lying Down*
7. Body Position Lying
Back
Side
8. Holding side rail? (# of hands)
Yes (1 or 2 hands)
No
9. Location in bed? When the patient lies down in the bed, is his/her head in the appropriate position so that the head is on the pillow?
Appropriate—yes
Inappropriate—no
If no, note if the patients is low in the bed, or too high (ie, Ss head on the head of the bed
10. Uses side rail to turn? (Number of hands)?
Yes (one or two hands?)
No
EGRESS
11. Does the subject use the rail to pull up into a sitting position?
Yes (One or two hands?)
No
12. Bounce to move down the bed? (Number of “bounces”)
Yes (How many?)
No
13. Hold side rail when turning at end of rail?
Yes
No
14. When the patient I sitting on the side of the bed, are the feet flat on the floor?
Yes
No, (give details)
15. Assisted to stand?
Yes (uses side rail, RN assist or walking aid?)
No
16. Does the patient use the side rail to maintain balance when standing?
Yes
No
***Standard Bed, side rails down* and *low bed***
1. Subject Number
INGRESS
2. What does the patient use for support when reaching the bed?
Hands—Hand flat on the mattress
Knuckles—clenched fist on the bed?
Fingers—Hand outstretched?
3. Nurse assist patient?
No-—Unassisted
Yes—Assisted by 1 (1) or (2) RNs?
4. Controlled or uncontrolled sit?
Controlled—Patient sits on bed in a without loosing balance or muscular control
Uncontrolled—Patient “flumps” or falls onto the bed in a sitting position
5. Leg effort (Assist) Is the patient able to lift his/her legs onto the bed without straining?
Yes—Legs lifted easily
No—Patient had difficulty in lifting legs onto the bed
(Assist)—if yes, were the legs lifted onto the bed by the RN? Yes/no?
6. Location in bed? When the patient lies down in the bed, is his/her head in the appropriate position so that s/he head is on the pillow?
Appropriate—yes
Inappropriate—no
If not, note if the patients is low in the bed, or too high (ie head on the head of the bed
7. Does the patient move up in the bed (That is pushing with hands on the mattress to lift buttocks, and pushing with heals to “bounce” up bed? (Number tries)
If yes, (Number of tries)
No
IN BED MOVEMENT
8. Body Position Lying
Back
Side
9. Pull on mattress to turn? Does the patient pull on the mattress to turn over?
Yes
No
EGRESS
10. Bounce to get out/assisted? (used mattress?) Does the patient lift him/her self across the mattress?
Yes (How many bounces?)
No
11. When the patient is sitting on the side of the bed, are the feet flat on the floor?
Yes
No, (give details)
12. Assisted to stand?
Yes (give details)
No
13. Required assistance when standing? Does the patient require support to maintain balance?
Yes
No

### Ethical Considerations

Permissions for the study were obtained from the University of Alberta, the University of Utah, and from the Alberta Health Authority, as well as the institutions involved. All participants were consented to the study and provided photographic releases. Participants were paid $25 CDN for participation, and reimbursed for expenses.

Appropriate fall protective devices (hip protectors) were provided for those with an impaired gait. Participants wore their “usual” footwear, and used their everyday walking aids (cane or walker). Registered nurses (RNs) provided assistance if needed, and they intervened when necessary. If the participant clearly had difficulty with sit-to-stand, assistance was provided.

## Results

Participants were recruited from a medical center acute care unit, two participants from nursing homes, and one normal control was recruited from the community. A total of 15 participants, 11 men (*M* age = 72.6 years) and 4 women (*M* age = 63 years) participated. Demographic information is presented in Table 1, including participant’s MFS scores, height, and weight. The results are organized as follows:

- A description of the participant with normal gait (Participant 1), ingress and egress, using the 23″ standard medical*-*surgical hospital bed, side rails up.
- A description of the observations of all participants using the 23″ standard medical*-*surgical hospital bed with the side rails up, as they ingress, turn over in bed, and egress.
- A description of the observations of all participants using the 23″ standard medical*-*surgical hospital bed with the side rails down, as they ingress, turn over, and egress; and
- A description of the observations of all participants as they ingress, turn over, and egress, using the 15″ mattress height low hospital bed, with the side rails down.

**Table 1.** Summary of the Participants’ Demographics.

Subject/ Site	Category/Disability/Dx	Gender	Age	Ht/Wt	MFS Score	Walking Aids/Assist	Floor Mat?	Footwear	Hip Protectors
1 Community	Normal	M	73	5′8″/138 lbs	0	None	No	None	No
2 NH1	Normal	M	84	5′6″/149 lbs	15	None	Yes	None	No
3 NH1	Parkinson disease	M	81	5′8″/168 lbs (est.) (too unstable to weigh or measure)	75	Walker	Yes	Shoes	Yes
4 NH1	Weak/impaired	M	78	5′7.5″/173.5 lbs	65	None	Yes	Slippers	No
5 Med Center	Surgical (bowel rotation and appendix)	F	23	5′4″/128 lbs	20	None	Yes	None	No
6 Med Center	Surgical	M	39	5′11″/176 lbs	20	None/IV pole	Yes	None	No
7 NH1	Not typical Parkinson disease. Walked well—Nurses report “freezes up,” and weak on left side	M	82	5′7″/183 lbs	65	None	Yes	None	Yes
8 Med Center	Surgical	M	69	5′10″/116 lbs	35	None/in pole	Yes	Socks	No
9 Med Center	Hemiplegia	M	59	5′7.5″/181 lbs	50	Cane	Yes	Slippers	Yes
10 NH1	Normal	F	73	5′0″/154 lbs	15	None	Yes	Gripper slippers	No
11 NH2	Hemiplegia (refused low bed trial)	F	66	5′5″/155 lbs (est.) too unstable to weigh or measure	75	Cane (prong base) Assist belt	No	Shoes	No (refused)
12 NH2	Parkinson disease	M	89	5′5.5″/158 lbs	35	None	Yes	Sneakers	No
13 NH2	Weak/impaired	F	90	4′8″/156 lbs	50	Walker (elbow rest)	No	Slippers	No
14 NH2	Weak/impaired	M	89	5′3″/191 lbs	40	Walker	No	Slippers	No
15 NH2	Parkinson disease (Advanced)	M	56	5′11″/154 lbs	75	Cane	No	Sneakers	No

*Note.* Ht = height; Wt = weight; MFS = Morse Fall Scale; IV = intravenous; SS# = Subject number;

NH = Nursing Home; NH1 = Nursing home 1; NH2 = Nursing Home 2.

Finally, we compare trials, and in the “Discussion,” provide Clinical Implications of the study.

### A Description of Normal Gait, Ingress, and Egress With the 23″ Mattress Height Standard Medical*-*Surgical Bed

#### Ingress, normal gait, 23″ mattress height

**Figure 2.**
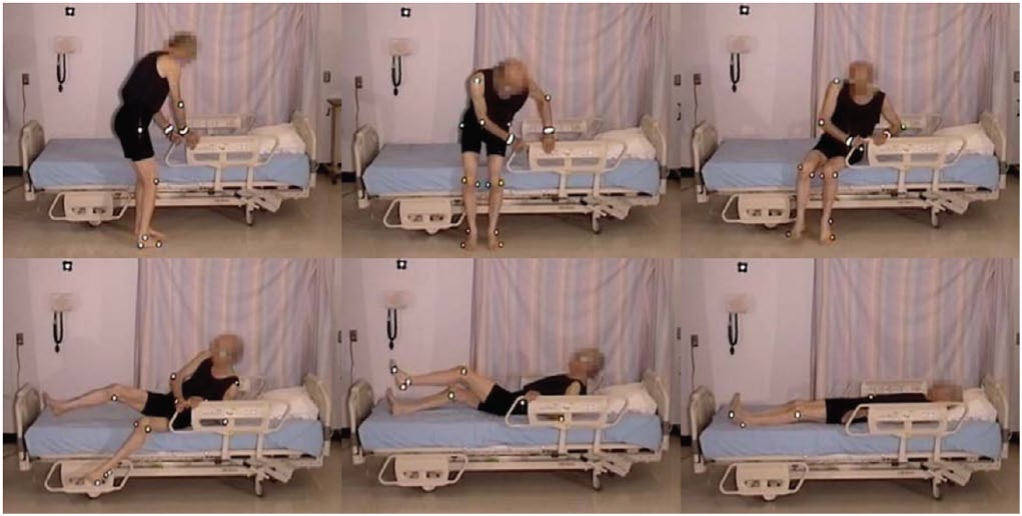
Normal gait, ingress.

An elderly participant with a normal gait (SS #1), walked to the bed, lifting his feet, and taking long strides. When reaching the bed, he stood facing the bed, and placed both hands on the side rail. Standing with his heels off the floor, he turned, and sat well back on the bed. Next, he leaned back and simultaneously swung his legs onto the bed. He used his elbows for support as he lay back on the bed on his back, with his body in the correct position with his head on the pillow (Figure 2, Video 1).

**Figure:**
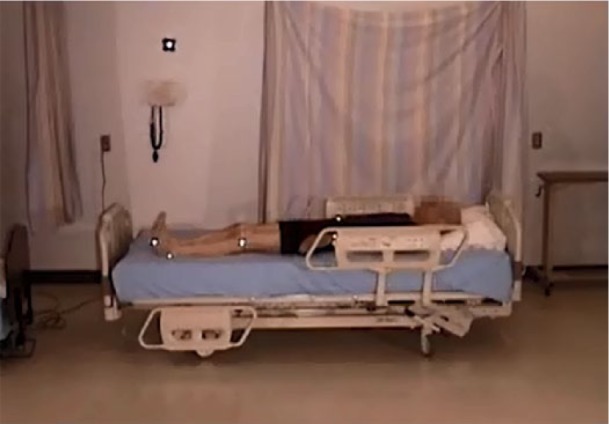


**Video 1.** Normal gait, ingress.

#### Egress, normal gait, 23″ mattress height

**Figure 3.**
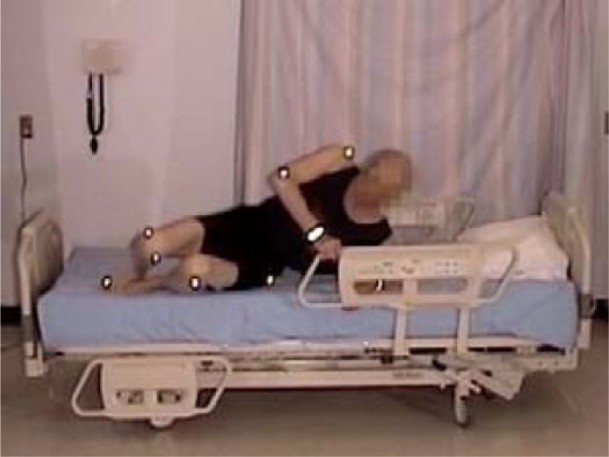
Hip hitch, egress.

The participant was lying on his side, and remained in on his side as he sat up. He grasped the side rail with his upper hand, pushed with his lower elbow, raising his upper body. Pivoting on his elbow, he used hip hitches to move down the bed, moving his hand along the side rail (see Figure 3 and Video 2). This hip hitch occurred when the participant was on his side, and then pushed on the bed with his top heel and lower elbow to lift his hips off the bed as he moved down the bed. As this participant sat up, he swung his legs out of the bed, pushing himself into an upright position with his hand remaining on the side rail. He then stood using both hands to support his stand. As soon as he was upright, he strode back to the chair.

**Figure:**
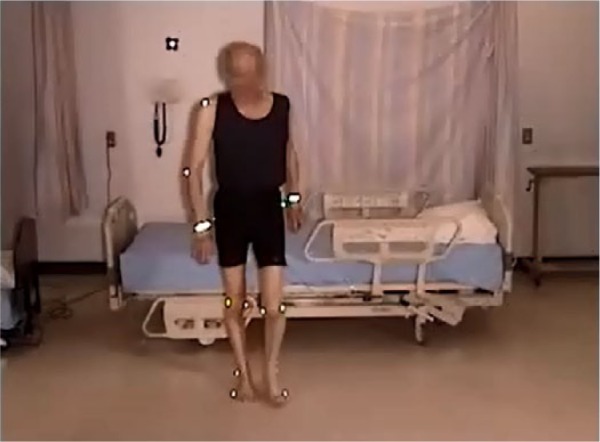


**Video 2.** Normal egress.

Note that the *hip hitch* (Natale et al., 2009) was also used with the participants in supine position, to move up or down the bed, or to turn over. With each hitch, they pushed on their heels and their hands to lift their pelvis off the bed. Then, they pushed with their feet to move up in the bed or with their arms to move down the bed.

### A Description of the Use of the 23″ Mattress Height, Side Rails Up

#### Side rails up, standard 23″ bed

Details of each participants’ movements for ingress, turning, and egress with side rails up are listed in Table 2. We observed participant stability (or imbalance) as he or she reached the bed and turned, their use of the side rails (and any assistance required), their control of sitting down on the bed, their ability to lift their legs into the bed, the location they selected to sit, and how they moved into position to lie down.

**Table 2.** Patient Activity With Standard 23″ Mattress Height Bed; Side Rails Up.

						In-Bed Movement				Egress					
	Ingress					Lying Down			Turning Over	Moving to Get Out			Standing		
Participant (Trial)	Hold Side Rail When Reaching Bed? (No. of Hands)	Nurse Assist?	Move Around Side Rail?	Effort to Lift Legs Onto Bed? Assisted?	Move Up in Bed? (Number of Tries)	Body Position Lying	Holding Side Rail? (No. of Hands on rail)	Location in Bed	Uses Side Rail? (No. of Hands)	Uses Rail to Pull to Sitting Position?	Scooting Move Down/Get Out Bed (No. of Tries)	Hold Side Rail to Turn off Bed?	Feet Flat on Floor When in Full Sit?	Pull Up? SR or Assist?	Holds SR When Standing for Support?
1 (4)	Yes (2)	No	Yes	No	Yes (1)	Back	No	Approp.	No	Yes (1)	Yes (1)	Yes (1)	Yes	Yes SR	No
2 (2)	No	No	Yes	No	Yes (2)	Back	No	Approp.	Yes (1)	No	No	No	No	No	No
3 (2)	No (walker)	1	No	Yes (yes)	Yes (1)	Side	No	Low	Does not turn	Yes (2)	Yes (1)	Yes (2)	Yes	Yes, 2 assist + walker	No
4 (3)	Yes (2)	No	Yes	No	No	Side	No	Approp.	Yes (1)	Yes (1)	Yes (1)	Yes (1)	Yes	Yes SR	Yes
5 (2)	Yes (1)	0	Yes	No	No	Back	Yes (1)	Approp.	Yes (1)	Yes (1)	Yes (1)	Yes (1)	Yes	No	No
6 (3)	Yes (1)	1	Yes	No	Yes (1)	Back	Yes (1)	Approp.	No	Yes (2)	No	Yes (1)	No	Yes SR	No
7 (3)	Yes (2)	0 (Entered frontward)	Yes	No	No	Back	Yes (2)	Approp.	Yes (2)	Yes (2)	Yes (2)	Yes (2)	Yes	Yes SR	Yes
8 (3)	Yes (1 + IV pole)	0	Yes	No	Yes (2)	Back	No	Approp.	No	No	Yes (1)	No	Yes	No	No
9 (2)	Yes (1)	0	Yes	No	Yes (1)	Side	No	Approp.	Yes (1)	Yes (1)	No	No (cane)	Yes	Yes (cane)	No
10 (2)	No	0	Yes	No	Yes (7)	Back	No	Approp.	No	No	Yes (4)	No	Leans Forward to Stand	No	No
11 (2)	No	One hand and one RN assist	No	Yes (yes)	No	Back	No	Low	Does not turn	Yes (one hand and two RN assist, behind and legs)	No	Yes (one hand and two RN assist)	No^a^	Yes SR, Cane and two RNs	Yes^a^
12 (3)	Yes (2)	0	Yes	No	Yes (1)	Back	Yes (1)	Low	Yes (2)	Yes (2)	No	Yes (2)	Yes	Yes SR (one hand)	Yes
13 (3)	No (walker)	0	Yes	Yes—Slight (no)	Yes (4)	Back	Yes (1)	Low	Yes (1)	Yes^a^ (2)	Yes (2)	No	No	No (push with knuckles)	No walker
14 (2)	No	0	No	Yes—Slight (no)	Yes (1)	Back	Yes (1)	Low	Yes (2)	Yes (2)	Yes (two with walker)	No	No	Yes walker	No walker
15 (1)	No	0	Yes	No	No	Back	No	Approp.	Yes (1)	Yes (1)	Yes (two with cane)	Yes (2)	Yes	Yes SR	Yes

*Note.* SR = side rails; Approp. = appropriate; RNs = registered nurses; IV = intravenous.

#### Moving toward the bed

**Figure 4.**
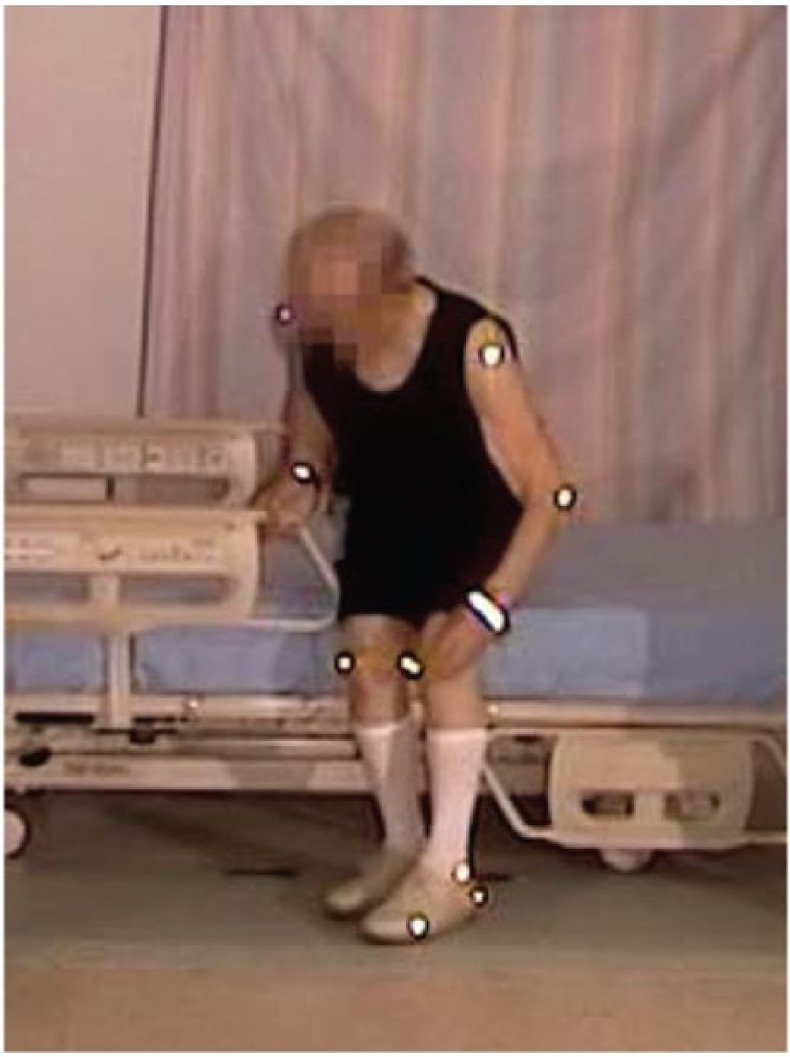
Using side rail for ingress.

Eight of the participants held onto the side rail when reaching the bed, using it to provide stability as they turned (Figure 4). This included the participant with a normal gait (Participant 1).

#### Ingress with the 23″ mattress height (standard bed), side rails up

All of the participants got into bed by sitting on the edge of the bed and then lifting their legs, except Participant 7, who entered the bed by climbing in front first. For this trial (the third trial for this participant), he entered the bed leaning forward on his knuckles, and then rocked back to sit. He “fell” to lie down on his back in an uncontrolled descent (see Video 3).

**Figure:**
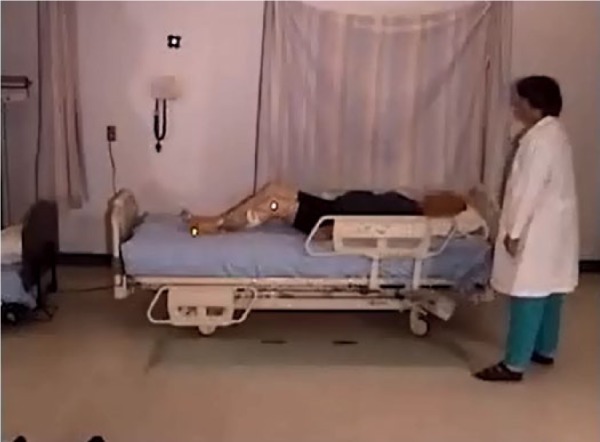


**Video 3.** Front first, ingress with uncontrolled descent.

#### Lifting legs onto bed

Once sitting on the bed, four of the participants had difficulty in lifting their legs onto the 23″ mattress height bed (Video 4), and two participants (Participants 3 and 11) required a nurse-assist to place their feet onto the bed. Ten participants used a hip hitch to move up in the bed, with one participant using 7 “bounces” including 3 hip hitches (Video 5)

**Figure:**
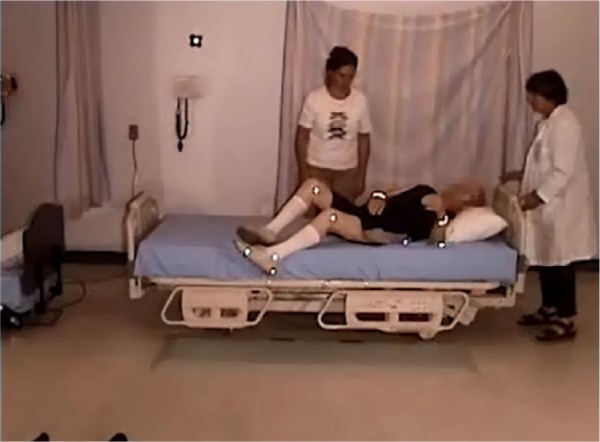


**Video 4.** Cannot lift legs onto bed.

**Figure:**
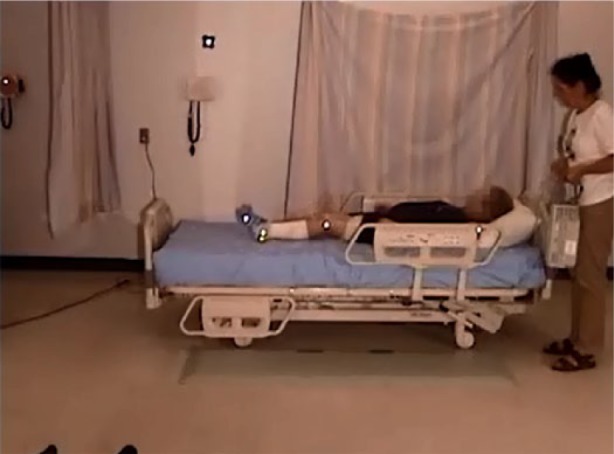


**Video 5.** Hip hitches to move up the bed.

The length of the raised side rails on the standard bed forced the participants to sit closer to the foot of the bed, and to move around the side rail to lie down. The handgrip on the side rails often provided poor support due to lateral movement (sway) of the side rail, and was apparently an awkward grip for support for moving up or down the bed.

Once in bed, some patients remained in a semi-sitting position. They used the hip hitch, pushing with their heels and their hands, until their bodies were high enough in the bed, so that when they lay down their heads would be on the pillow. Using hip hitches to move up the bed required exertion: Participant 3 adjusted her position as she sat back in the bed, and then she used three bounces to move up in the bed (see Video 5). If the participant chose not to move up in bed, his or her body was positioned close to the end of the bed.

Three participants elected to lie down on their sides (without holding onto the rails), with the remainder of the participants lying down on their backs (with 6 using one or both hands on the rails). When asked to turn onto their sides, rails were used by 9 of the 13 participants, who turned pulling with one hand, and with 3 participants using both hands. This indicates that the side rails provided assistance (and were used) for in-bed mobility by 12 participants.

#### Egress with the 23 mattress height (standard bed), side rails up

Exiting the bed with the upper rails raised involved 12 of the participants using the rails to pull themselves into a semi-sitting position. Video 6 demonstrates this process. Most (*n* = 10) then moved their buttocks down the bed using a hip hitch movement, while using the side rails for support. Again, participants had to “move around the rail” to exit. Video 7 demonstrates this process, and shows that the lower edge of the rail may even become an obstacle. Nine participants held the rail as they turned to sit on the edge of the bed, 7 used the rail as they stood, and 5 held the side rail for support as they stood, regaining their balance before walking back to the chair.

**Figure:**
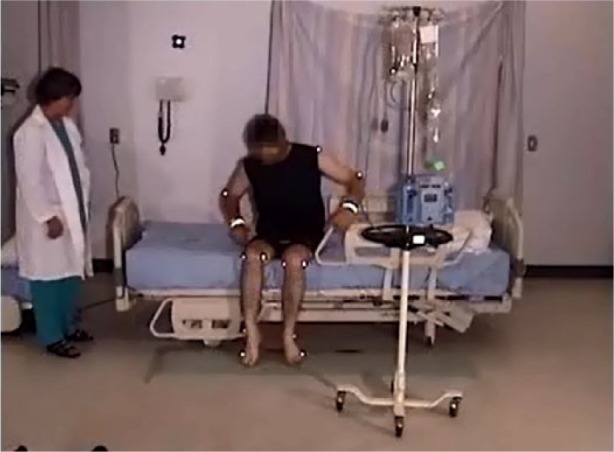


**Video 6.** A semi-seated position to egress

**Figure:**
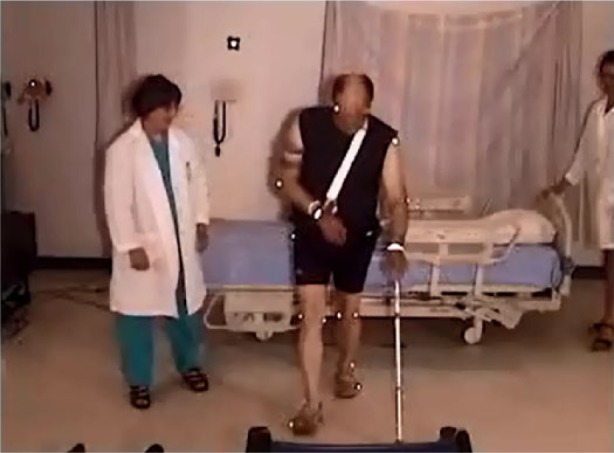


**Video 7.** Egress around raised side rail.

**Figure 5.**
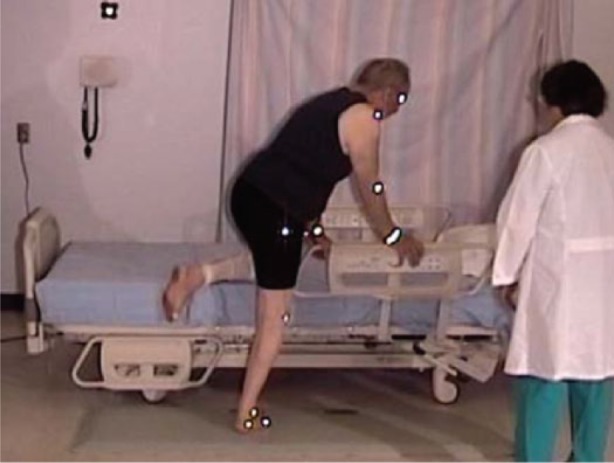
Front first, ingress, uncontrolled.

### A Description of the Use of the 23″ Mattress Height, Side Rails Down

#### Ingress, side rails down

**Figure 6.**
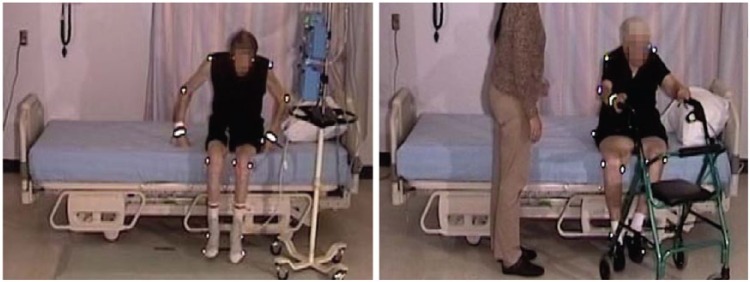
Participants sitting too high on the bed.

As they reached the bed, when the side rails were lowered, patients were more likely to extend their fingers and lean on the mattress, or use their knuckles for support. One participant required assistance getting into bed (see Table 3). Ingress appeared easier without the side rails, as they did not have to move around the side rail to lie down. However, they did not show good judgment about where they sat on the mattress. Rather than tending to lie toward the foot of the bed, two participants lay down too high in the bed. These patients consistently selected positions to sit on the bed that were too close to the head of the bed, necessitating that they hip hitch down the bed (or be lifted by the staff), so that their head was on the pillow (Figure 6). Patients with an impaired gait were apparently unable to judge the optimal locations to sit when approaching, turning, and sitting on the bed.

**Table 3.** Patient Activity With Standard Bed; Side Rails Down.

Participant (Trial)	Ingress						In-Bed Movement		Egress			
	Support With Hands/Knuckles?	Nurse Assist?	Controlled/Uncontrolled Sit?	Leg Effort? (Assist?)	Location in Bed	Move Up in Bed? (No. of Tries)	Body Position Lying	Pull on Mattress to Turn?	Scooting to Get Out/Assist? No. of Bounces (Used Mattress?)	Sit—Both Feet Flat on Floor	RN Assist to Stand?	Required Assist When Standing?
1 (2)	One hand, one knuckles	No	Controlled	No	Approp.	No	Back	No	Yes, one bounce (yes)	Yes	No	No
2 (3)	One hand	No	Controlled	No	Low	Yes (2)	Back	No	No (knuckles)	One foot down	No	No
3 (1)	Walker	One assist	Controlled (with assist)	Yes (no)	Approp.	No	Side	Yes	Yes, one bounce	No	One assist + Walker	Yes
4 (1)	Two hands with fingers	No	Controlled	No	Approp.	No	Back	No	Yes, two bounces (knuckles)	Yes	No	No
5 (1)	One hand with fingers	No	Controlled	No	Approp.	Yes (1)	Back	No	No (no)	Yes	No	No
6 (2)	Two knuckles	No	Controlled	No	Approp.	No	Back	No	Yes, one bounce (yes)	Yes	No	No
7 (2)	Knuckles	No	Controlled	No	Approp.	Yes (1)	Back	Did not turn	Yes, one bounce (no)	Yes	No	No
8 (1)	Knuckles	No	Controlled	No	High	Down (1)	Back	No	No (no)	Yes	No	No (unsteady)
9 (1)	Cane	No	Controlled	No	Approp.	No	Back	No	No (no)	Yes	Cane	No
10 (1)	No	No	Controlled	No	Approp.	Yes (2)	Back	No	Yes, one bounce (no)	No	No	No
11 (1)	One hand and one RN assist	One assist	Controlled	Yes (no)	Backward	Moved pillow	Back	Did not turn	No (no)	One foot down	Yes (2) and cane	x2 assist
12 (2)	One hand, one knuckle	No	Controlled (feet off floor)	Yes (yes—Legs lifted)	Approp.	No	Back	No	No (no)	No	No	x2 assist with balance
13 (1)	Walker	No	Controlled (feet off floor)	Yes (no)	High	No	Back	Tries to turn, pulls on head of bed—Did not turn	Yes, one bounce (no)	No	No walker	No walker
14 (1)	One hand	No	Controlled	No	Approp.	No	Back	No	No (no)	Yes	No walker	No walker
15 (3)	One hand, one cane	No	Controlled	No	Approp.	No	Back	No	Yes, two bounce (no)	Yes	No	No

*Note.* Approp. = appropriate; RNs = registered nurses. x2 = Twice.

### A Description of the Use of the Low Bed 15″ Mattress Height, No Rails

#### Ingress with low bed, no side rails

The low bed was trialed without side rails. This bed, with a mattress height of 15″, was higher than the low-low beds presently available, which have a deck height of 9.5″. Even so, one participant (Participant 11, hemiplegia, MFS score 75) refused this part of the trial because it was “too low.”

As participants approached the bed and bent to sit, they reached back for the mattress with their hands (3 participants with one hand; 1 with one hands) or knuckles (4 participants with one hand and 2 with two hands). Two used a walker and one used a cane to lower themselves onto the bed (Table 4). This was rated as an “uncontrolled descent” (sit) in 4 of the 14 participants, and 1 participant was assisted descent. Those with an uncontrolled descent were 3 (Parkinson Disease, MFS 75), 4 (weak gait, MFS 65), 7 (Parkinson Disease, MFS 65), and 13 (MFS 50). Of these participants, 1 participant with Parkinson Disease, rolled backward across the bed, and his lower legs were caught by a researcher (see Video 8). The bed was so low that another participant’s walker was unable to provide support, and he experienced an uncontrolled descent, and the walker fell with him (see Video 9, floor camera). None of the participants had difficulty lifting their legs onto the low bed.

**Table 4.** Patient Activity With Low Bed; Side rails down.

Participant (Trial)	Ingress						In-Bed Movement		Egress			
	Support With Hands/Knuckles?	Nurse Assist?	Controlled/Uncontrolled Sit	Leg Effort? (Assist?)	Location in Bed	Move Up in Bed? (No. of Tries)	Body Position Lying	Pull on Mattress to Turn/Sit?	Scooting (No.) to Get Out/Assist?	Sit—Feet Flat on Floor	Assist to Stand?	Required Assist When Standing?
1 (1)	One hand	No	Controlled	No	Approp.	No	Back	No	No	Yes	No	No
2 (1)	One hand	No	Controlled	No	Approp.	No	Back	No	No	Yes	No	No
3 (3)	Walker	Two assists	Uncontrolled	No	Low	Yes (4)	Side	Does not turn/no	Yes (3)	Yes	Yes, three bounces; two assist	Yes, two assists
4 (2)	Two hands	No	Uncontrolled	No	Approp.	No	Side → Back	No	Side → Pushes off mattress with knuckles (4)	Yes	No	No
5 (3)	One knuckle	No	Controlled	No	Approp.	No	Back	No	No	Yes	No	No
6 (1)	Two knuckles	No	Controlled	No	Approp.	No	Back	No	Yes (1)	Yes*	No	No
7 (1)	One knuckle	No	Uncontrolled	No	Approp.	No	Side*	No	Yes (4)/x2 assist	Yes	Yes x2 assists	Yes x2 assists
8 (2)	One knuckle	No	Controlled	No	High	Yes (1)	Back	No	No	Yes	No	No
9 (3)	Cane	No	Controlled	No	Approp.	No	Back	No	Yes (2)	Yes	No cane	No
10 (3)	Two knuckles	No	Controlled	No	Low	Yes (2)	Back	No	Yes (3)	Yes	No	No
11	Refused trial											
12 (1)	One hand	No	Controlled	No	Approp.	No	Back	Did not turn	Yes (3)/one RN assist	Yes	Yes. two hands push, three bounces, one RN	Yes
13 (2)	One knuckle	No	Uncontrolled	No	High	No	Back	Yes, grabs low rail	Yes (3)/two RN assists	Yes	Yes x2 assists + Walker	Yes, two assists then to walker
14 (3)	Walker	No	Controlled	No	Low	No	Back	Did not turn/no	Yes (1)/one RN assist	Yes	Yes one assist + Walker	Walker
15 (2)	Cane	No	Controlled	No	Approp.	No	Back	No	No	Yes	Yes, two assists	Yes

*Note.* Approp. = appropriate; RNs = registered nurses. x2 = Twice, *= Superscript.

**Figure:**
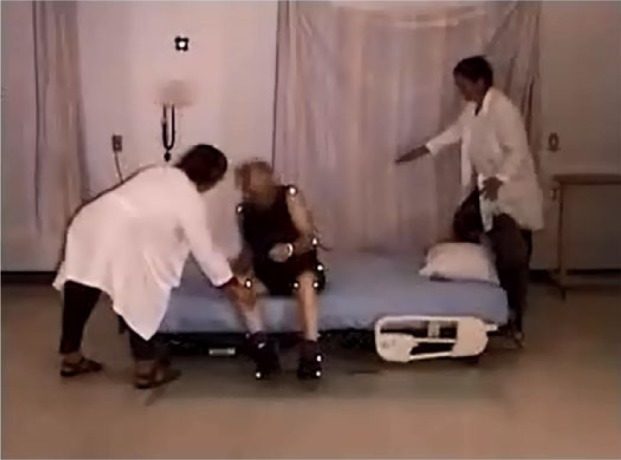


**Video 8.** Save from fall, low bed.

**Figure:**
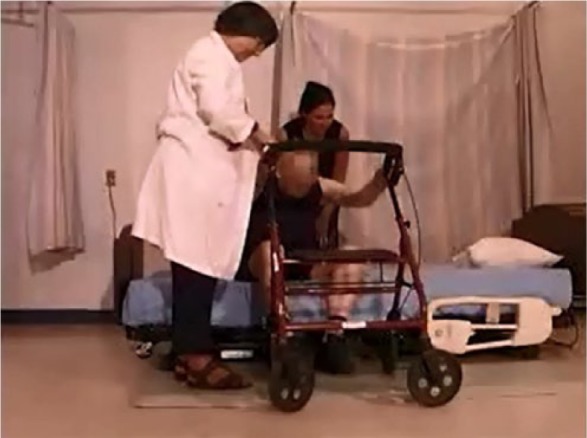


**Video 9.** Walker not supportive, ingress low bed.

**Figure 7.**
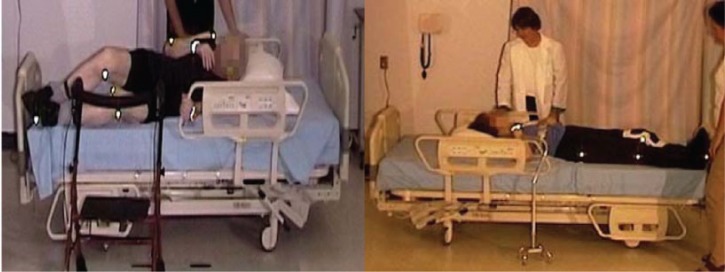
Participant is too low in the bed.

**Figure 8.**
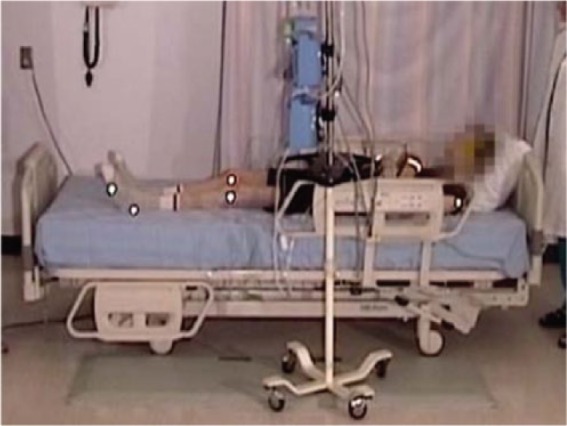
Participant is too high in the bed.

The participant’s location in the bed when lying down was rated as “appropriate” (i.e., head on pillow) in 9 participants; 3 were too high in the bed (Figure 7) and 3 were too low (see Figure 8). Three participants moved about in the bed, adjusting themselves using a hip hitch, from 1 to 4 times; 11 participants did not adjust their position or were lying in an appropriate location.

All but three participants turned over in bed. One was unable to pull himself on his side and leaned over the bed and reached to use the lowered rail to pull himself over (see Video 10).

**Figure:**
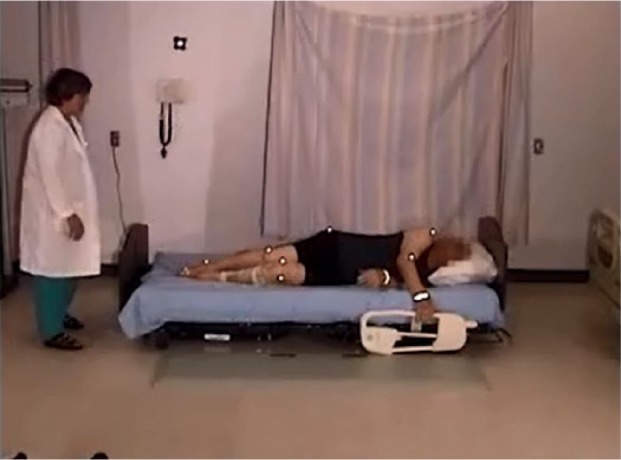


**Video 10.** Participant pulling lowered rail to turn.

#### Egress with low bed, no side rails

**Figure 9.**
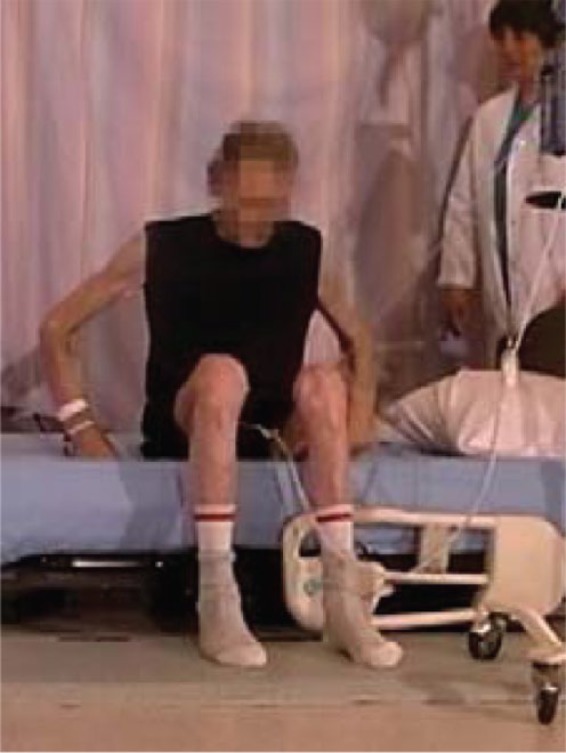
Tall participant, low bed.

Four participants used a hip hitch to get out of the bed (from 1 to 4 times), and required the assistance of two nurses (see Video 11). All participants were able to sit comfortably on the side of the bed with their feet flat on the floor. However, for a tall participant, the bed was uncomfortably low and his knees high (see Figure 9).

**Figure:**
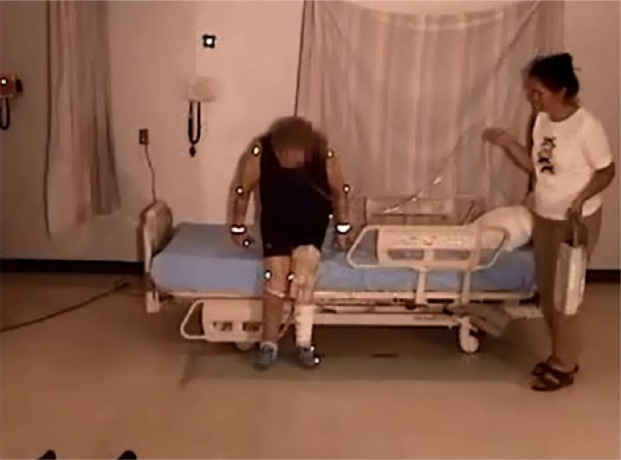


**Video 11.** Using a hip hitch to move down the bed.

Standing was difficult from this low mattress height, and it was accomplished without assistance by only 7 of the 15 participants (with 1 using a cane), with 5 participants requiring one or two assistants for sit-to-stand. There were two problems in rising: the effort required to raise from the low height as well as poor balance when standing. Once standing, 2 participants required assistance to regain balance, and 1 used a walker (Video 12).

**Figure:**
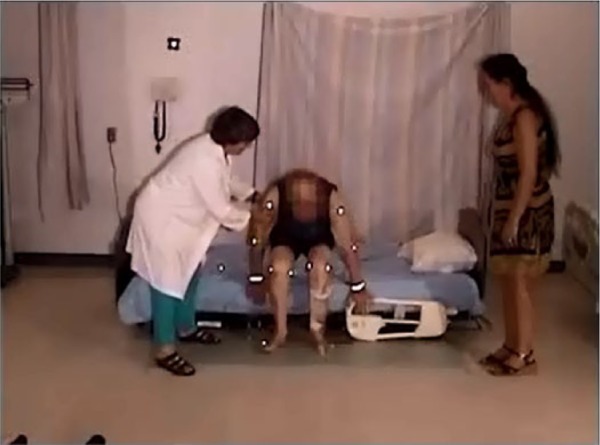


**Video 12.** Sit-to-stand from low bed.

### A Comparison of Trials

From the above analysis, answers to the specific research questions and recommendations will be presented. With the small sample size, it is not our intention to make statements with any degree of statistical certainty, but rather to highlight observed indicators with clinical implications, or which require further investigation. To address the main research question, the sub-questions will be addressed first.

#### How are the side rails used for in-bed mobility, and do they enable the patient to lie down, to sit up, or to turn over in bed more easily? Side rails were used by all but 2 of the 15 patients for some activity

Eight used the side rails when turning to sit on the bed, 5 when they were lying down, 9 when they were turning over, 10 pulled up on the side rail to climb off the bed, 5 used it to regain their balance once standing. From this, we conclude that side rails are used by some patients to facilitate bed entry and exit and for in-bed movement. The length of the upper side rail inhibited bed entry and exit for weaker patients, and did not allow for appropriate body location when lying without lifting oneself up in the bed.

#### Do the side rails provide a stable handhold, thereby facilitating bed entry and a more stable gait when exiting the bed? In this study, it appeared that those patients who had obvious problems with balance used the side rails for support

We could not, however, evaluate the grip on the side rails. It was evident that the side rails had considerable “play” (i.e., lateral movement) and it appeared that participants did not perceive the side rails to be a secure handhold. All of the patients who had MFS scored over 45 (with the exception of Participant 4), had either a cane or walker, which, if they were also being assisted, excluded them from grasping the rail as they entered or exited the bed. Nevertheless, the frequency of the use of the rails when moving about the bed indicated their usefulness.

The shape of the side rail, however, was not always ergonomically optimal for grasping. When pulling up from a lying position, the end of the rail was too low for a comfortable reach and not useful until the participant moved down the bed. The angle was also not optimal if the patient was standing, and the top of the rail was too low for tall persons to provide adequate support.

#### Is the bed height (mattress height) a factor influencing the ease of entering and exiting the bed and the stability of the gait? Initially, we were concerned that the low bed provided for the trial had a 14″ or 15″ bed height (depending on the presence of a floor mat)

Given that the lowest beds on the market presently may have less than a 10″ mattress height, we were initially concerned that there would not be an adequate height difference between the 15″ bed height and the bed with the 23″ mattress height to make an observable clinical difference in this trial. Nevertheless, in this trial, all but the most agile patients had problems sitting on the low bed, and there was one “incident” in which the patient rolled across the bed and was caught before he fell off the other side. All of the participants maintained a controlled descent getting into the 23″ mattress height bed, compared with 11 participants in the low bed—plus 4 “uncontrolled” descents. For two of the three participants who used a walker for support when sitting on the bed, the bed was so low that the walker tipped and failed to provide safe support as the participant sat. Participants could rise from the low bed only with effort (or with great effort) or with assistance, and on rising had difficulty in keeping their balance with nothing to hold onto. (A more comprehensive analysis of the bed height is reported elsewhere, Merryweather et al., in press.)

We conclude that the height of the low bed was unsafe, unless used consistently with a pole or nursing assistance. The low bed should only be used in the clinical setting following observation of the patient entering and exiting, to assess that the bed is safe for that particular patient. The clear advantage of the low bed—to prevent injury should a patient roll out of bed—was not tested in this study. However, the recommendation that the “height of bed frames should be specified to be as low as possible” (Tzeng & Yin, 2005, p. 325, 2007, p. 358) is inaccurate and unsafe, and is not based on patient performance.

However, the bed with the 23″ mattress height appeared too high for all patients when entering the bed. Some patients were not able to sit with their buttocks completely on the bed with their feet on the floor, or were supporting themselves with only their toes. All but one participant climbed into bed by correctly first sitting on the edge of the bed. As the low bed trial was conducted first on the participant who subsequently entered both of the 23”mattress height trials by climbing into bed front first, it is possible his decision not to climb in by sitting on the bed was based on his incident with the low bed. The fact that this style of getting into bed (front first) was used by only one participant does not discount the fact that other patients may attempt to climb into bed front first, and this style should be considered with care. However, Tzeng and Yin’s (2008) recommendation that all patients should be taught to enter the bed using the prone position, should be examined for slip potential, before recommending this as standard practice.

The higher mattress height showed that overall participants could rise more easily. But getting out of the 23″ mattress height bed remained too high for short, elderly participants. One patient moved to the edge of the bed trying to reach the floor, and was considered to be at risk of falling front first onto the floor. Some participants reached down to the floor with one leg—a strategy that assists with maintaining balance but increases risk of slipping off the mattress. While six participants required RN assistance when rising from the low bed, only three participants required assistance from the 23″ mattress height bed without side rails, and only one participant required assistance with side rails up. Thus, it appeared that the side rails increased stability and ease of exiting from a higher seating position, but for short participants, the risk of slipping onto the floor must also be considered.

From this study, we conclude that *all* patients cannot safely ingress or egress in a hospital bed. The low bed was more difficult, therefore “less safe,” than the standard height bed. Turning over in bed was difficult for some patients. We recommend that additional research regarding side rails and bed height be conducted.

## Discussion

As these data were collected in 2003, beds have been developed with variable mattress heights, and some offer controlled rising of the deck to assist with standing. Furthermore, side rail design has changed on newer models, so that the top rail is shorter, and “moving around the rail” for ingress or egress is less difficult. However, as many institutions continue to use the models of beds used in this study, the findings remain pertinent and significant.

In this study, although the sample size did not represent all disabilities of all patients at risk, the variation of participants was adequate to demonstrate problems with bed design that placed patients at risk of fall. Although videotaping in the hospital setting may produce more naturalistic results, it is not feasible. Studies such as these must be conducted in a laboratory, with adequate lighting and the space for all the necessary cameras.

From this study, the following findings are suggested:

A. Side rails were used by participants to aid entry, in-bed movement and bed exiting

When turning in bed, the side rails were used by 10 participants to pull and assist with in-bed mobility; 2 were unable to turn, and only 3 turned without the rails (Table 2). When the rails were not in position, 2 pulled on the mattress or the head of the bed, and 2 were unable to turn over. Given the significance of in-bed mobility, this is an important use of side rails.

B. If the top side rail extends mid-way along the deck (or further), it will impede the bed entry and exit of the patient

Patients must move around the rail and up and down the bed. This action poses the patient at risk of skin damage to the sacrum and hips, and if the patient is too weak to move up the bed, requires staff lifting and places staff at risk of back injuries. More recent models have reduced the length of the top rail, so that when the patient is sitting on the side of the bed and lies down, his or her head is on the pillow. A second lower rail (or another partial rail) is installed on some models for patients who require longer rails (but *not* rails that are the full length of the bed). Full-length side rails do not provide a safe route from the bed, and should never be used.

C. No side rails

When the side rails were down, participants did not have a handhold for support as they entered or exited the bed. Two participants even reached over the side of the bed searching for a handhold to pull themselves onto their sides.

One unexpected finding was that the side rail served as a visual cue when getting into bed. It served as a “target” that indicated participants with an impaired gait should sit on the bed. In this study, with this bed, the rail was too long and the position not optimal, and the participant lay down on the bed too close to the foot of the bed. When the rail was down, participants selected any position on the side of the bed to sit, and to lie down, and often this was too close to the head of the bed. Both of these events resulted in considerable effort for patients to move up or down the bed to put their head on the pillow. If it was necessary for the staff to lift the patient into position with the patient’s head on the pillow, this placed the staff at risk of back injury, or the patient at risk of a skin “shearing” injury, with subsequent risk of pressure ulcers. With beds that have the top side-rail positioned so that patients may situate their buttocks in a position, so that when they lie down they are positioned correctly in the bed, both patients and staff safety would be enhanced, with considerable cost savings to hospitals and the health care system. Patients with an impaired gait had difficulty in judging the optimal place to sit on the side of the bed, so they would be able to lie with their head on the pillow: This was an unexpected finding. In Scandinavia, there is an indicator of impaired gait, “stops talking when walking” (Lundin-Olsson, Nyberg, & Gustafson, 1997). It is possible that for participants who have an impaired gait, the effort of walking requires such concentration to walk, that the elderly may not have the ability both to walk and to cognitively determine specific placement on the side of the bed during ingress. One possible intervention would be to place a stripe across the center of the sheets, to provide the patients with a visual cue to show them where to sit when getting into bed.

D. Bed height

One clear finding is that, as patients are of different heights and abilities, then ideally the bed height should be adjustable to the patients’ needs, so that the bed “fits” the patient. Entering low beds is dangerous for patients with limited hip flexion, and increased effort is needed to rise out of the low bed. This is particularly difficult for patients with impaired balance, and additional assistance is needed from staff. This study confirms the findings of Capezuti et al. (2008) that the 23″ mattress height bed, even in its lowest position, was too high for some patients when entering.

The difference in performance of participants entering and exiting the bed suggests that two deck heights are required: one for safe ingress and a different height for egress. It is unrealistic to retain the present model of one deck height “fits all,” and a safety goal must be the development of a bed that is adjusted to “fit” the patient, providing automatic “reset” to the predetermined safest height for ingress or egress.

### Limitations

In this study, we did not measure patient leg strength or arm strength, and were unable to correlate participant strength measures with ability to lift legs onto the bed and to calculate the forces required on the side rails during ingress, turning, and egress. Also, we did not have adequate luminous markers to evaluate balance and gait from an ergonomic perspective, hence the observational nature of this study. Was the sample size of 15 patients adequate? We used theoretical maximum variation sampling, but for Institutional Review Board (IRB) consent concerns, excluded the cognitively impaired patient. Yet regardless of patients’ cognitive status (and their inability to follow instructions to “stay in bed”), it remains our responsibility to provide a bed that is as safe as possible for ingress and egress, and we must find a way to explore bed safety within this group. Future studies should also determine the forces required on the side rails during ingress, turning, and egress. It is recommended that the study be replicated, to evaluate bed height and side rails according to patients’ disabilities.

## Conclusion

While the physical characteristics of patients who fall have been extensively investigated, factors related to the hospital bed (from which they frequently fall) has not been investigated. From this study, it was determined that not all elderly and disabled patients can safely enter, exit and move about a standard medical/surgical hospital bed. We showed that all patients were not able to lift their legs onto the bed. Side rails, when they extended beyond the midpoint of the bed, became an obstacle to move around, forcing the patient to scoot up the bed, or resulted in the patient lying down too close to the end of the bed. When side rails were removed, patients were unable to judge where they should sit on the bed, and sometimes lay down too high in the bed. When turning in bed, most used side rails to assist with the maneuver. The clinical implications of these findings are summarized in Box 1.

Box 1. **Clinical implications**

**Clinical implications**

**Deck height:** As it is usual standard protocol to maintain occupied beds in the low position, it is recommended that staff assess the patient’s ability to move in and out of bed. For some patients, the low position may not be the safest position, and they may require assistance either to get into bed, or out of bed, or required the bed to be slightly higher than the low setting.

**Low beds:** These are intended to reduce the distance of the top of the mattress and the floor, and therefore reduce injury if a patient should roll out of bed. However, this study shows that even beds with 15inch mattress height were unsafe for getting in and out.

Ingress: Assess the patient’s ability to safely enter the bed. Patients with an inflexible hip joint may experience an “uncontrolled sit.”

Egress: Assess the patient’s ability to rise from the bed. The patient may lack strength or the balance necessary to stand. Once standing, often there is nothing to hold onto. If a low-low bed must be used (perhaps because of patient confusions and restlessness) it is recommended that a bed alarm also be used so that the staff know when the patient rises from the bed. A walker should also be placed at the bedside.

**Side rails**

As side rails have been redesigned, and the risk of entrapment has been removed. From this observational study, it is recommended that side rails be used to assist with patient mobility:

Inbed: Side rails assist with turning, and lifting self up and down the bed.

Ingress: Side rails assist with the standing turn-to-sit, and provide a stable handhold when sitting on the side of the bed.

Top side rails, assist the patient with an impaired gait to correctly position their buttocks as they turn to sit on the side of the bed, so that when they lie down, their head is on the pillow.

Egress: Side rails are used to enhance balance with the patient sits to stand, and begins to move away from the bed.

Haines et al. (2010) noted that low-low beds were used for two reasons: (a) to minimize injury, should the patient roll from the bed (and therefore also reduce the need for restraints); and (b) to “limit the ability of the cognitively confused impaired patient to stand from the bed, and therefore not place themselves at risk of falling” (pp. 435–436). We conclude that low-low beds, while they may reduce injury if the patient rolls out of bed (this was not explored in this study), may actually cause injury on ingress and egress. The cognitively impaired patient may attempt to stand, and because of impaired balance and the lack of a handhold, fall. Furthermore, on ingress, patients who lack hip flexibility will “fall” onto the low bed in an uncontrolled descent, which may also result in an incident as they roll over the bed and out the other side.

In this study, the side rails appeared to be an asset, enhancing ingress, in-bed mobility, and egress. Side rails manufactured for use on hospital beds have been redesigned and are no longer a threat to patient entrapment. No longer considering side rails as a risk, Healey, Oliver, Milne, and Connelly (2008) now attribute this problem to the “use of outmoded designs and incorrect assembly” (p. 368). Full-length side rails are not used. Always having a safe route out of the bed reduces the possibility of a patient climbing over the rails or over the end of the bed. As these design threats to patients safety have now been corrected, it is recommended that side rails be reintroduced to increase in-bed mobility, and safe ingress and egress. Furthermore, Healey et al. (2008) suggest that research examining the effect of side rails is “uninformed by a current and comprehensive critique of the empirical evidence on bedrails” (p. 369) on increasing falls and injury severity is inconclusive. Two cohort studies located in Healey et al.’s (2008) review found “no significant difference in fall rates with or without bed rails” (p. 375). Our present study had a different focus. Rather than investigating side rail falls over the top of the rail, we explored side rails as a necessary assistant for mobility. We recommend that side rails be used clinically for the support of in-bed mobility, ingress, and egress, and this approach must be further investigated.

Presently, because all beds have many features in common, the implicit assumption is that all beds are safe for all patients. In this study, we attempted to replicate clinical conditions. Our patients were selected because of differing gait and problems, but were generally considered “mobile” without nursing assistance. Yet, the standard bed height was too high for safe egress for all patients. The side rails or walking aides were used as support for sit-to-stand. Patients were unable to sit with control on ingress with the low bed (which is 4.5″ “higher” than the deck of the low-low bed now available), and resulted in a near incidents with two patients in uncontrolled descents. When egressing, patients were unable to sit-to-stand and maintain balance; six participants required assistance to stand and one patient refused the trial.

All beds were not safe for all patients.

This study was not a test of a specific bed, but rather, an examination of performance of hospital beds with specifications that are shared by many manufacturers. Of importance were the ideas regarding bed height: that beds do not need to be adjusted to meet individual patient needs, and that the bed in the low position is optimal for patient safety. Our study revealed that the low position may not be the safest position for all patients. Yet, we are aware that even if the low bed height position could be adjusted to meet patients’ needs, two problems remain: (a) we must counter the present belief and recommendation that, when occupied, all beds must be in the low position must be abandoned; and (b) if there is an ideal individualized bed height according to patient ability and stature, a determination must be made as to what that height is, how to calculate it, and how to modify the design of beds, so that bed height may be individualized accordingly. Such an advance would greatly reduce patient falls.

Patient injury has reached epidemic proportions, and the iatrogenic environment in hospitals: In particular, the risks for patients when entering or exiting the hospital bed must be addressed. Nursing has the main responsibility for fall prevention, both in vigilance and in monitoring (Shaw, Connelly, & McWilliam, 2014), and also in implementing best practice guidelines (Boblin, Ireland, Kirkpatrick, & Robertson, 2013). It is time to extend the responsibility of preventing patient falls beyond nursing surveillance, and to involve researchers with expertise in human movement analysis, biomechanics, and ergonomics to develop safe environments to prevent patient falls.

## Supplementary Material

Supplementary material

## Supplementary Material

Supplementary material

## Supplementary Material

Supplementary material

## Supplementary Material

Supplementary material

## Supplementary Material

Supplementary material

## Supplementary Material

Supplementary material

## Supplementary Material

Supplementary material

## Supplementary Material

Supplementary material

## Supplementary Material

Supplementary material

## Supplementary Material

Supplementary material

## Supplementary Material

Supplementary material

## Supplementary Material

Supplementary material

## Notes

1. The bed was a Hill-RomTM Advanta©. The deck height is unchanged with the new models; the top side rail was shortened and redesigned with the Advanta2©, which has three split rails on each side.

2. Since this study was conducted, a low-low bed is now available with a deck height as low as 9.5″. This does not discount the findings of the low bed in this study—we must assume that the low-low beds extend these findings, increasing the risk to patients.
